# Applying a mobile intervention for chronic insomnia in routine care: Study protocol for a multicenter randomized controlled trial

**DOI:** 10.1016/j.invent.2025.100848

**Published:** 2025-06-20

**Authors:** Daa Un Moon, Jeonghun Kim, Jeyoung Hannah Sun, Yujin Lee

**Affiliations:** a Department of Psychiatry and Neurosciences, Charité Campus Mitte, Charité Universitätsmedizin Berlin, Corporate Member of Freie Universität and Humboldt-Universität zu Berlin, Berlin, Germany; b Department of Consultation-Liaison Psychiatry and Psychosomatic Medicine, University Hospital Zurich, University of Zurich, Zurich, Switzerland; c WELT Corp. Ltd., Seoul, Republic of Korea; d College of Pharmacy, Yonsei Institute of Pharmaceutical Sciences, Yonsei University, Incheon, Republic of Korea

**Keywords:** Insomnia, Chronic insomnia, Digital cognitive behavioral therapy, CBT-I, Mobile health, Digital intervention, Digital therapeutics, mHealth

## Abstract

Cognitive behavioral therapy for insomnia (CBT-I) is the first-line treatment for chronic insomnia. However, access to in-person CBT-I remains limited due to a shortage of trained providers and structural barriers. Digital CBT-I (dCBT-I) offers a scalable solution to bridge this treatment gap, yet real-world evidence of its effectiveness remains limited. This study aims to examine the effectiveness of a mobile dCBT-I intervention, “SleepQ,” in a routine clinical setting. This study is a multicenter, two-arm, open-label, randomized controlled trial comparing a mobile dCBT-I intervention to a waitlist control group. A total of 120 adults with chronic insomnia will be recruited from six clinics in South Korea. Participants will be randomly assigned (1:1) to either the intervention group receiving 6 weeks of dCBT-I or the control group, which will gain access to the intervention after the posttreatment assessment. The primary outcome is the Insomnia Severity Index score change from baseline to 6 weeks post-randomization. Secondary outcomes include dysfunctional sleep beliefs, daytime sleepiness, depressive and anxiety symptoms, quality of life, and work productivity. Exploratory outcomes include adherence, usability, app satisfaction, and sleep parameters from the integrated sleep diary. In the intervention group, follow-up assessments will be conducted 3- and 6-months post-randomization to evaluate long-term effects. This trial will evaluate the effectiveness of mobile dCBT-I for chronic insomnia within routine clinical care. Findings will contribute to the evaluation of the clinical implementation of digital therapeutics for insomnia and inform the integration of mobile-based CBT-I into routine care.

**Clinical trial registration:**

https://clinicaltrials.gov/study/NCT06695000, ClinicalTrials.gov (NCT06695000).

## Highlights

• Cognitive behavioral therapy for insomnia (CBT-I) is the first-line treatment.
• Digital CBT-I (dCBT-I) delivers treatment via mobile application.
• This multicenter RCT evaluates the effectiveness of dCBT-I in routine care.

## 1 Introduction

Chronic insomnia is a sleep disorder characterized by difficulty falling asleep, staying asleep, or experiencing non-restorative sleep, leading to daytime fatigue and impaired daily functioning despite adequate opportunity for sleep (American Psychiatric Association D, Association AP, 2013; World Health O, 1992). It is distinguished from acute insomnia, which typically lasts days to a few weeks, by its persistence: diagnostic criteria generally require symptoms to persist for a minimum of 1 to 3 months, depending on the classification system (American Psychiatric Association D, Association AP, 2013; Organization WH, 1992).

Chronic insomnia is a prevalent condition, affecting approximately 10–15 % of adults in industrialized nations (Ohayon and Reynolds III, 2009), with reported rates in South Korea reaching 14 % in recent years (Ahn et al., 2024). The disorder often follows a chronic course, with epidemiological studies estimating that over half of affected individuals experience persistent symptoms for several years (Morin et al., 2009). Beyond its impact on sleep, chronic insomnia is linked with increased risks of psychiatric disorders, cardiovascular diseases, metabolic syndrome, and reduced quality of life (Léger et al., 2012; Zhang et al., 2021; Markwald et al., 2013; Malik et al., 2014; Breslau et al., 1996; Baglioni et al., 2011; Neckelmann et al., 2007; Mayer et al., 2011; Yaffe et al., 2014). Furthermore, the disorder carries societal and economic consequences, contributing to reduced workplace productivity, increased absenteeism, elevated healthcare utilization, and a higher risk of accidents (Sivertsen et al., 2009a; Sivertsen et al., 2009b; Daley et al., 2009).

Given its widespread impact, effective treatment options for insomnia are essential. Cognitive behavioral therapy for insomnia (CBT—I) is the gold-standard treatment recommended by international guidelines as the first-line approach for managing insomnia across all age groups (Riemann et al., 2023a; Chung, 2019; Qaseem et al., 2016). CBT-I utilizes a structured therapeutic framework, incorporating behavioral strategies such as stimulus control and bedtime restriction, along with cognitive techniques aimed at modifying dysfunctional thoughts about sleep (Krystal et al., 2019; Van Straten et al., 2018). Importantly, CBT-I targets the perpetuating factors of insomnia, such as maladaptive sleep habits, dysfunctional beliefs, and conditioned arousal, that sustain chronic insomnia beyond its initial precipitating events. Multiple studies have confirmed its long-term effectiveness in improving sleep outcomes, with benefits extending beyond symptom management to reducing reliance on pharmacological treatments (Koffel et al., 2015; Wu et al., 2015; Smith et al., 2002; Edinger et al., 2001; Backhaus et al., 2001). Although medications such as benzodiazepine receptor agonists or sedating antidepressants are frequently prescribed for short-term relief, they are associated with potential side effects, including dependence, cognitive impairment, and increased risk of falls in older populations (Moon et al., 2024; De Crescenzo et al., 2022; Andrade, 2018; Riemann and Perlis, 2009). As a result, their long-term use is generally discouraged in clinical guidelines (Chung, 2019; Riemann et al., 2023b; Sateia et al., 2017).

Despite the proven efficacy of CBT—I, access to treatment remains a major challenge (Moon et al., 2024). Structural barriers such as the limited availability of trained therapists, long waiting times, and geographic constraints hinder the widespread implementation of in-person CBT-I (Thomas et al., 2016; Rossman, 2019; Fields et al., 2013). In response to these challenges, digital CBT-I (dCBT-I) has emerged as an innovative and scalable approach to delivering evidence-based insomnia treatment via mobile applications or web-based platforms. DCBT-I programs integrate interactive tools such as sleep diaries, psychoeducation, and structured cognitive-behavioral interventions (Soh et al., 2020; Zachariae et al., 2016; van der Zweerde et al., 2020). Several studies have demonstrated the efficacy of dCBT-I in improving sleep outcomes (Soh et al., 2020; Zachariae et al., 2016; Ebert et al., 2018; Espie et al., 2019; Lorenz et al., 2019; Luik et al., 2017). As a result, regulatory bodies in several countries, including Germany, the United Kingdom, and the USA, have approved dCBT-I programs for clinical use (Lorenz et al., 2019; Behrendt et al., 2020; Morin, 2020; Digital Therapeutics Alliance, n.d.). Current clinical guidelines recognize dCBT-I as an evidence-based treatment, highlighting its potential to bridge the treatment gap in sleep medicine (Riemann et al., 2023b).

Despite its growing adoption, real-world evidence of its effectiveness as an adjunct to routine clinical care remains limited (Dreyer, 2018; Ritterband et al., 2022). In South Korea, a mobile-based dCBT-I program has been approved by the Ministry of Food and Drug Safety (MFDS) as a prescription digital therapeutic (PDT) (Lee, 2020; Shin et al., 2024). “SleepQ”, the MFDS-approved intervention, was developed through a multidisciplinary process involving clinicians, engineers, and behavioral scientists to ensure clinical and technical robustness. Its clinical validity was evaluated in a randomized controlled trial conducted under regulatory conditions. In addition, a pilot study evaluating SleepQ in combination with wearable sleep-tracking technology has been conducted to inform future integration and scalability. While regulatory approval by the MFDS confirms the clinical validity of mobile dCBT-I, its integration and effectiveness within routine clinical care remain largely untested. Most existing trials have evaluated dCBT-I under controlled conditions, limiting generalizability to real-world clinical practice. This study directly addresses this gap by assessing the effectiveness of SleepQ as an adjunct to usual care in clinical settings, thereby generating real-world evidence for implementation in routine care.

This study protocol outlines a multi-center randomized controlled trial (RCT) designed to evaluate the effectiveness of an MFDS-approved dCBT-I intervention compared to a waitlist control group. The findings will contribute to understanding the real-world applicability of dCBT-I in routine clinical settings and its potential role in bridging the treatment gap for chronic insomnia.

## 2 Methods

### 2.1 Objectives and hypotheses

This study aims to evaluate the effectiveness of the mobile self-help application “SleepQ” as an adjunct to standard care for individuals with chronic insomnia in routine outpatient settings, within a superiority trial framework. The intervention is based on dCBT-I and is designed to address core insomnia symptoms through structured behavioral and cognitive strategies. We hypothesize that participants receiving dCBT-I in addition to standard care will show greater reductions in self-reported insomnia severity compared to the waitlist control group (CG) after 6 weeks of intervention.

As secondary objectives, we hypothesize that dCBT-I will lead to greater improvements across several additional domains compared to the CG. Specifically, we expect that participants in the intervention group will report greater reductions in maladaptive sleep beliefs, daytime sleepiness, and depressive and anxiety symptoms, along with improved work productivity and health-related quality of life. Furthermore, we will assess the stability of treatment effects in the intervention group at 3- and 6-month follow-ups. Exploratory analyses will examine app-based sleep diary parameters, user adherence, and engagement with the intervention.

### 2.2 Participants and recruitment

The study will be conducted across six clinics located throughout South Korea (Supplementary Table 1). These clinical sites were selected to ensure geographic and institutional diversity and to reflect routine clinical care conditions. In addition to supporting participant recruitment, the clinics serve as locations for conducting in-person assessments at baseline, post-treatment and follow-up time points. A total of 120 participants with chronic insomnia will be recruited using multiple channels, including clinic bulletin boards, clinic websites, printed flyers in clinics, and personal recommendations. Interested individuals will be invited to an in-person screening session to receive detailed study information and provide written informed consent before any study-related procedures. Eligibility will be assessed by a board-certified psychiatrist or neurologist, reviewing the participant's medical and medication history and conducting a general health examination to determine suitability for study inclusion. Participants in both the intervention and control groups will receive USD 120 (paid in KRW equivalent) upon completion of the post-treatment assessment at 6 weeks. This amount covers compensation for participation in all study procedures from baseline (t0) through post-treatment (t3). Additionally, participants in the intervention group will receive USD 15 for each completed follow-up assessment at 3 months (t4) and 6 months (t5), totaling up to USD 150 for full participation. Compensation will be provided upon completion of each respective assessment.

#### 2.2.1 Eligibility criteria

Adults aged 19 years or older, with no upper age limit, who meet the ICD-10 criteria for chronic insomnia (F51, G47) and have experienced insomnia symptoms for at least three months will be eligible to participate in this study. Participants must have an Insomnia Severity Index (ISI) score of ≥8, be proficient in Korean, own a smartphone with internet access, and be able to use mobile applications independently.

Individuals with coexisting sleep disorders, such as obstructive sleep apnea, parasomnia, or restless legs syndrome, will be excluded. These will be screened based on clinical interviews, medical history, and symptom profiles conducted by a board-certified psychiatrist or neurologist. Participants whose symptoms suggest a primary sleep disorder other than insomnia will not be eligible. Those who have adjusted their medication regimen within the past three months for drugs that may affect sleep—including hypnotics, sedatives, antidepressants, anxiolytics, anticonvulsants, or antipsychotics—will also not be eligible. To prevent systematic pre-treatment group differences regarding healthcare service utilization and ensure that differences in outcome changes can be attributed to the intervention, participants who have undergone non-pharmacological insomnia treatments, such as psychotherapy or light therapy, within the past three months will also be excluded. Additional exclusion criteria include pregnancy or plans for pregnancy during the study period, shift work, or engagement in activities where sleep deprivation poses a safety risk. Individuals with severe medical conditions, including active or progressive physical illnesses (e.g., congestive heart failure, chronic obstructive pulmonary disease, or acute pain), neurological conditions (e.g., stroke, epilepsy), neurodegenerative diseases (e.g., dementia, multiple sclerosis), or suicidality, as well as those with unstable health and a life expectancy of less than six months, will not be eligible. To ensure that the study reflects routine clinical care, individuals diagnosed with insomnia who also meet the criteria for other mental disorders will be included.

### 2.3 Trial design

**Fig. 1:**
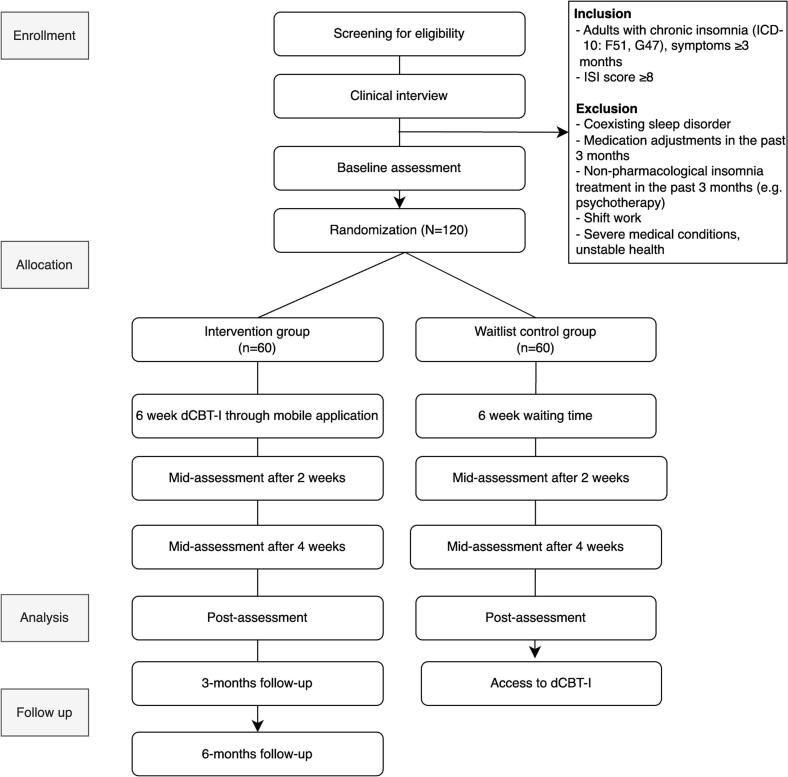
CONSORT Diagram of Study Enrollment Flow.

This study is a multicenter, two-arm, open-label RCT designed to evaluate the effectiveness of a dCBT-I intervention. The trial follows the Standard Protocol Items: Recommendations for Interventional Trials (SPIRIT) 2013 guideline (Chan et al., 2013). The multicenter approach, involving six outpatient psychiatric clinics across South Korea, was chosen to enhance the generalizability across diverse clinical settings. Eligible participants will be randomized into either the intervention group (IG) or the waitlist control group (CG) in a 1:1 allocation ratio. The IG will receive dCBT-I (SleepQ, WELT Corp. Ltd) over 6 weeks. The CG will receive no intervention from the study team during this period but will be given access to dCBT-I upon completion of the posttreatment assessment at 6 weeks post-randomization. The waitlist design enables short-term comparison while ensuring all participants ultimately receive access to the intervention. Additionally, participants in the intervention group will be followed up at 3- and 6 months post-randomization (Fig. 1). Due to the behavioral nature of the intervention, participant blinding is not feasible. However, allocation will be concealed during statistical analysis to minimize potential bias.

Ethical approval was obtained from each clinic's Institutional Review Board (IRB), and the trial was preregistered on clinicaltrials.gov. The trial will be conducted in compliance with the ethical principles outlined in the Declaration of Helsinki. Written informed consent will be obtained from all participants prior to enrollment. Participation in the study is voluntary, and participant will be informed of their right to withdraw from the study at any time without providing a reason.

#### 2.3.1 Randomization and blinding

Participants meeting the eligibility criteria will be randomized in a 1:1 allocation by an independent researcher not involved in the study. A stratified block randomization approach will be applied, stratifying participants based on the study site and sleep medication use. Randomization will be performed via an Interactive Web-Response System, which will generate computer-randomized sequences with concealed allocation. Diagnostic interviewers conducting clinical assessments will remain unaware of participants' group assignments. After randomization, each participant will immediately receive an access code for the intervention (IG) or the information that the intervention will start in 6 weeks (CG). Due to the study design, blinding of participants and study personnel to group allocation during the intervention period is not feasible.

Group allocation will be masked by an independent researcher, who will anonymize datasets by removing identifying allocation variables before statistical procedures. Consequently, evaluators will remain blinded to intervention and control conditions throughout the analysis.

#### 2.3.2 Intervention

The dCBT-I intervention will be delivered via a mobile application designed to provide a fully automated, interactive, and tailored experience. The development of the app involved a multidisciplinary team, including clinicians, software developers, product designers, and behavioral scientists, to ensure both clinical validity and user accessibility. The app will feature a modular structure, incorporating educational content, self-monitoring tools, interactive exercises, goal-setting capabilities, relaxation techniques, and a frequently asked questions (FAQ) section. The content will be presented in various formats, including informative texts, audio files, videos, and interactive quizzes. The CBT-I modules will be delivered sequentially, guiding participants through a structured therapeutic process: psychoeducation, stress models and relaxation techniques, bedtime restriction, sleep hygiene education, stimulus control, cognitive restructuring, and relapse prevention(Table 1). Participants will have access to various relaxation techniques, including autogenic training, progressive muscle relaxation, autogenic training, and mindfulness exercises. These techniques will be available in different formats and durations, allowing users to select methods that best match their individual needs and levels of tension. Participants will complete a digital sleep diary integrated into the app interface, based on standardized parameters recommended in the Korean clinical guideline for insomnia (Chung, 2019). Users will input daily variables such as bedtime, wake time, time in bed, sleep onset latency, and wake after sleep onset. The app will automatically calculate derived sleep metrics (e.g., sleep efficiency) and provide personalized feedback to support bedtime restriction. A virtual guide named “SSAM”, designed as a professional female doctor in a white coat, will guide participants through the program by providing instructions, feedback, and encouragement. The name SSAM is derived from the Korean word for “teacher”, a term commonly used as a respectful way to address medical professionals. Participants will be encouraged to use the app at their convenience, typically spending 10 to 15 min per day engaging with the content and completing exercises. The app includes built-in features designed to promote consistent engagement, such as a visual progress tracker and automated usage reminders. The virtual guide “SSAM” offers instructional prompts and encouragement throughout the program to support adherence.

**Table 1:** Overview of Digital CBT-I Modules and Their Therapeutic Components.

	Modules	Description
1	Introduction and Goal setting	Introduction to the intervention, setting personalized goals, and outlining the therapy structure.
2	Psychoeducation	Explanation of sleep physiology, sleep cycles, and the role of cognitive and behavioral factors in insomnia.
3	Stress management & relaxation techniques	Theoretical background on stress and its impact on sleep, with an introduction to relaxation exercises such as breathing techniques and progressive muscle relaxation.
4	Bedtime restriction	Regulation of sleep-wake timing to enhance sleep efficiency, including setting a personalized sleep window based on sleep diary data.
5	Half time check in	Review of individual progress, reflection on sleep patterns, and adjustment of goals based on initial experiences.
5	Sleep hygiene education	Strategies to optimize the sleep environment and daily habits that influence sleep quality, including light exposure, caffeine intake, and bedtime routines.
6	Stimulus control	Behavioral techniques to strengthen the association between bed and sleep, reducing time spent awake in bed
7	Cognitive interventions	Identification and restructuring of dysfunctional sleep-related beliefs, with emphasis on reducing nighttime rumination and anxiety.
8	Relapse prevention	Strategies to maintain long-term sleep improvements, including identifying early warning signs of insomnia relapse and implementing coping strategies.

The mobile application is available for both Android and iOS devices. It has been approved as a PDT by the Korean MFDS in April 2023. To maintain data security and privacy, the application adheres to the cybersecurity guidelines established by the MFDS (Division, 2023). All collected data will be securely stored on a cloud-based server, with encryption measures to protect participant information. Additionally, the development and operation of the application will comply with Good Manufacturing Practice standards, ensuring the digital intervention's quality, safety, and reliability (Safety, 2025). Access to the application will require verification through the Korean identity verification (IDV) system, ensuring only authorized participants can engage with the intervention.

#### 2.3.3 Control group

Individuals allocated to the waitlist control group will not receive access to the intervention during the 6-week study period. After completing the final assessment at 6 weeks post-randomization, participants in the control group will be provided access to the dCBT-I intervention.

#### 2.3.4 Concomitant care

Participants who are already undergoing treatment with sleep medications, antidepressants, anxiolytics, anticonvulsants, sedatives, or antipsychotics at a stable dosage before enrollment will be able to continue their medication during the study. To maintain consistency in treatment conditions, participants will be instructed to follow their existing prescription without modifying the dosage or frequency during the study period. Additionally, the use of medications prescribed for treating other medical conditions will be recorded. If participants require new medications during the study period, including those for newly occurring conditions or adverse events, relevant details such as the drug name, indication, dosage, start date, and end date will be documented. To ensure that study outcomes reflect the effectiveness of the digital intervention, participants will be informed not to engage in additional structured sleep interventions, such as CBT—I, during the study period.

### 2.4 Measures

Assessments will take place at screening/baseline (t0), 2 weeks after baseline (t1), 4 weeks after baseline (t2), and 6 weeks after baseline (t3, post-treatment). Additionally, participants in the IG will undergo follow-up assessments at 3 months (t4) and 6 months (t5) post-randomization. Assessments at t1, t2, t4, and t5 will be conducted remotely, while screening (t0) and post-treatment (t3) assessments will take place in person. An overview of the clinical outcome measures over time, based on the Standard Protocol Items: Recommendations for Interventional Trials (SPIRIT) 2013 guidelines, is provided in Table 2.

**Table 2:** Schedule of enrolment, interventions, and assessments according to SPIRIT.

	STUDY PERIOD						
Study period	Enrolment	Allocation	Post-allocation			Follow up	
Timepoint	*-t*_*1*_	*t*_*0*_	*t*_*1*_	*t*_*2*_	*t*_*3*_	*t*_*4*_	*t*_*5*_
Week		0	*2*	*4*	*6*	*12*	*24*
Enrolment							
Informed consent	X						
Eligibility screen	X						
Clinical Interviews	X						
Allocation		X					
Interventions							
Intervention Group: Digital cognitive behavioral therapy for insomnia		X	X	X	X		
Control Group: Waitlist control		X	X	X	X		
Assessments							
Demographic and clinical information		X					
Primary outcome							
ISI		X	X	X	X	X^a^	X^a^
Secondary outcomes							
ESS		X	X	X	X	X^a^	X^a^
DBAS-16		X	X	X	X	X^a^	X^a^
PHQ-9		X	X	X	X	X^a^	X^a^
GAD-7		X	X	X	X	X^a^	X^a^
SF-36		X	X	X	X		X^a^
WPAI-SHP		X	X	X	X		X^a^
Other measures							
Sleep parameters from sleep diary: SE, TST, SOL, WASO			X^a^	X^a^	X^a^		
SUS					X^a^		
App satisfaction questionnaire					X^a^		
App usage compliance					X^a^		
Adverse event			X	X	X		
Concomitant medication assessment			X	X	X	X^a^	X^a^
Concomitant therapy assessment			X	X	X	X^a^	X^a^

Abbreviations: ISI, Insomnia Severity Index; ESS, Epworth Sleepiness Scale; DBAS-16, Dysfunctional Beliefs and Attitudes about Sleep-16; PHQ-9, Patient Health Questionnaire-9; GAD-7, Generalized Anxiety Disorder-7; SF-36, Short Form-36; WPAI-SHP, Work Productivity and Activity Impairment Questionnaire–Specific Health Problem; SUS, System Usability Scale; SE, sleep efficiency; TST, total sleep time; SOL, sleep onset latency; WASO, wake after sleep onset.

a Only administered to intervention-group.

#### 2.4.1 Primary outcome

The ISI was selected as the primary outcome measure based on evidence from previous meta-analyses and studies with similar designs (Behrendt et al., 2020; Schuffelen et al., 2023; Ritterband et al., 2009). This 7-item self-report questionnaire assesses insomnia severity over two weeks, covering sleep difficulties, dissatisfaction, impairment, and distress. Scores range from 0 to 28, with higher scores indicating greater severity. Score ranges correspond to the following severity levels: 0–7 (no clinically significant insomnia), 8–14 (subthreshold insomnia), 15–21 (moderate insomnia), and 22–28 (severe insomnia) (Cho et al., 2014; Bastien et al., 2001; Morin et al., 2011).

#### 2.4.2 Secondary outcomes

The Epworth Sleepiness Scale (ESS) measures daytime sleepiness by evaluating the likelihood of falling asleep in eight daily situations (Johns, 1991; Cho et al., 2011). Each item is rated on a 4-point scale, yielding a total score ranging from 0 to 24. Higher scores indicate greater daytime sleep propensity. Scores are interpreted as follows: 0–10 (normal), 11–12 (mild), 13–15 (moderate), and 16–24 (severe) daytime sleepiness. Although individuals with chronic insomnia typically report fatigue and cognitive tiredness rather than excessive physiological sleepiness, the ESS was included to explore potential improvements in perceived daytime functioning following treatment.

The Dysfunctional Beliefs and Attitudes about Sleep (DBAS-16) assesses maladaptive sleep-related cognitions (Morin et al., 2007; Yu et al., 2009). It consists of 16 items, each rated on a 10-point scale, with higher scores reflecting stronger dysfunctional beliefs about sleep.

The Patient Health Questionnaire-9 (PHQ-9) evaluates depressive symptoms over the past two weeks (Martin et al., 2006; Park et al., 2010). It consists of nine items, each scored on a 4-point scale, with higher scores indicating greater severity. Total scores range from 0 to 27, categorized as follows: 0–4 (none/minimal), 5–9 (mild), 10–14 (moderate), 15–19 (moderately severe), and 20–27 (severe depression).

The Generalized Anxiety Disorder-7 (GAD-7) assesses generalized anxiety symptoms over the past two weeks. It includes seven items, rated on a 4-point scale, with higher scores indicating greater severity. Total scores range from 0 to 21 and are interpreted as: 0–4 (minimal), 5–9 (mild), 10–14 (moderate), and 15–21 (severe anxiety) (Spitzer et al., 2006; Lee et al., 2022).

The Short Form-36 (SF-36) measures health-related quality of life across eight domains, including physical functioning, mental health, social functioning, and general well-being (*J Health Info Stat.*, 2003; Ware Jr. and Sherbourne, 1992). Scores range from 0 to 100, with higher scores indicating better health status.

The Work Productivity and Activity Impairment Questionnaire–Specific Health Problem (WPAI-SHP) evaluates the impact of insomnia on work productivity and daily activities. It consists of four subscales: absenteeism, presenteeism, overall work impairment, and daily activity impairment (Reilly et al., 1993). Higher scores reflect greater impairment in work and daily functioning.

#### 2.4.3 Exploratory outcomes

Exploratory outcomes will be assessed only in the IG.

The SleepQ mobile application includes a daily sleep diary feature, allowing participants to log sleep efficiency (SE), total sleep time (TST), sleep onset latency (SOL), and wake after sleep onset (WASO).

The system usability scale (SUS) is a 10-item standardized questionnaire that assesses the usability and user experience of the SleepQ platform (Brooke, 1996). Scores range from 0 to 100, with higher scores indicating greater perceived usability. Participants' satisfaction with the app will also be assessed using a self-designed questionnaire that evaluates acceptability, feasibility, and overall satisfaction with the intervention.

Adherence to the intervention will be measured using log files from the SleepQ platform, which capture the number of logins, dates of access, and completed modules. These data will help determine engagement patterns and intervention compliance.

#### 2.4.4 Other outcomes

Data on gender, date of birth, education level, occupation, religion, marital status, residential setting, income level, smoking status, and alcohol consumption will be collected through participant self-report during the baseline assessment. Medical history, including pre-existing conditions and surgeries within the past year, current medication use, and prior treatments for insomnia or related conditions, will also be recorded.

All adverse events reported during the study will be documented in the electronic case report form. Details recorded will include the type of event, onset and end date, severity, any interventions required, outcomes, and the assessed causal relationship to the medical device.

Throughout the study, information on concomitant medications and non-pharmacological therapies will be collected. The data will include the medication's name, indication, dosage, frequency, start and end dates, and any modifications.

### 2.5 Data management, monitoring, and risk management

All research staff will receive training on the study protocol to ensure standardized trial execution.

Participant data will be pseudonymized using unique identification codes and stored in encrypted, password-protected systems. A secure and access-restricted linkage file (participant key) will be maintained separately from study data and stored on a password-protected server at the coordinating institution. Only authorized members of the study team will have access to this key for the purpose of responding to participant requests or managing withdrawals.

Study data will be stored in encrypted, password-protected systems and regularly reviewed for accuracy. Investigators will monitor study progress and data completeness, with annual audits conducted by the trial data manager. Given the low-risk nature of the intervention, no formal data monitoring committee will be established.

Participants may request access to, correction of, or deletion of their personal data at any point during the study period and up to four weeks following their final participation, in accordance with the Korean Personal Information Protection Act. For statistical analysis and dissemination, the dataset will be fully anonymized and no longer traceable to individual participants.

As a non-invasive digital intervention, SleepQ is considered low-risk; however, some participants may experience temporary psychological discomfort. Psychological assessments at study time points will monitor distress, with referrals to clinical care provided if needed. Participants who withdraw from the study will be asked to provide reasons, and follow-up data collection will continue where possible. Trial insurance covers potential adverse effects, though no significant risks are anticipated. Protocol amendments will require IRB approval and will be reported in the published article.

### 2.6 Planned statistical analysis

The analysis will include participants following the full analysis set based on the Intention-to-Treat principle, which includes all randomized participants who have completed a baseline assessment of the primary outcome (ISI score). All statistical analyses will be performed using SAS Software Version 9.4 or higher, with a two-sided significance level of 0.05.

Descriptive statistics will summarize baseline demographic and clinical characteristics, adherence, and usability measures. Depending on their distribution, continuous variables will be reported as means and standard deviations or medians and interquartile ranges, while categorical variables will be presented as frequencies and percentages.

To evaluate the primary outcome at post-treatment (t3, 6 weeks), an Analysis of Covariance (ANCOVA) will be conducted, adjusting for baseline values and stratification factors (study site and sleep medication use) as covariates to compare differences between the IG and the CG. Effect sizes (Cohen's d) will be reported alongside *p*-values and confidence intervals to indicate the magnitude of between-group differences. A mixed-effects model for repeated measures (MMRM) will be applied to assess longitudinal treatment effects. This model considers three repeated assessments (t1, t2, t3) as nested within individuals, accounting for intra-individual correlations and missing data. No imputation of missing values is planned, as MMRM accommodates missing data under the assumption that data are missing at random. A stepwise modeling approach will be used, beginning with a random intercept model without predictors. Next, fixed effects for time (study entrance, 2 weeks, 4 weeks) and treatment (IG vs. CG) will be added. Finally, a treatment × time interaction term will be introduced to examine whether outcome trajectories differ between groups. Treatment effects will be reported using Cohen's d as a measure of effect size. Descriptive analyses will be conducted to explore patterns of missingness, including comparisons of baseline characteristics between study completers and non-completers, as well as the timing and extent of dropout across timepoints.

Additionally, sleep diary data will be analyzed to explore trends in sleep parameters. Weekly mean values for sleep efficiency (SE), total sleep time (TST), sleep onset latency (SOL), and wake after sleep onset (WASO) will be calculated. An additional MMRM analysis will examine trends in these sleep parameters over time. Post hoc exploratory analyses may also examine potential effect modification by baseline characteristics, such as sleep medication use, alcohol consumption, and smoking status.

### 2.7 Statistical power and sample size

The sample size was determined using G*Power 3.1 (Faul et al., 2007), based on an ANCOVA with a fixed-effects model, assuming a large effect size (Cohen's f = 0.485) derived from previous research, which reported heterogeneous effect sizes ranging from 0.97 to 2.08 (Cohen's d) (Behrendt et al., 2020; Schuffelen et al., 2023). Cohen's f is the conventional effect size metric for ANCOVA models, and can be derived from Cohen's d. The calculation was conducted with an alpha level of 0.05, a statistical power (1- beta) of 0.90, and 3 covariates, including baseline scores and stratification factors. The G*Power analysis indicated that a total sample size of 97 participants would be required to achieve the desired power. The required sample size was adjusted to account for a 20 % attrition rate, resulting in a final target of 120 participants.

## 3 Discussion

This study protocol outlines a multicenter RCT evaluating the effectiveness of a mobile-based dCBT-I intervention compared to a waitlist control group. The intervention, approved as a PDT by the Korean MFDS, aims to address barriers to access to insomnia treatment by providing a scalable and evidence-based solution. The study will assess the effects of short-term (6 weeks) and long-term (3 and 6 months) treatment using validated patient-reported outcomes, including the ISI as the primary outcome. Secondary outcomes will evaluate changes in maladaptive sleep beliefs, daytime sleepiness, depressive and anxiety symptoms, health-related quality of life, and work productivity. Additionally, exploratory analyses will examine intervention adherence, usability, and app satisfaction, contributing to a better understanding of dCBT-I engagement in routine care.

This trial has several methodological strengths. It is one of the first RCTs evaluating an MFDS-approved dCBT-I intervention in real-world clinical settings, increasing its clinical relevance. The multicenter design enhances generalizability across different healthcare settings. Including both short-term and long-term follow-up assessments allows for evaluating treatment durability beyond immediate symptom improvements. Sleep diary data and application usage metrics also complement self-reported outcomes, comprehensively evaluating intervention effects. The study's broad eligibility criteria improve generalizability to routine clinical settings (Schuffelen et al., 2023; Maurer et al., n.d.).

Despite its strengths, the study has several limitations. Due to the open-label design, participants will be aware of their group allocation, which may introduce expectation bias. However, blinding was not feasible given the nature of the intervention, and data analysis will be conducted by an independent researcher blinded to group allocation to minimize potential bias. Another limitation is the use of a waitlist control group instead of an active comparator, which does not account for placebo effects or non-specific therapeutic effects. Future research should compare dCBT-I to in-person CBT-I or pharmacological treatments to determine comparative effectiveness (Kallestad et al., 2021).

Furthermore, participant engagement with the intervention may vary, and adherence challenges could impact treatment outcomes. To address this, intervention adherence will be monitored using app log data, and dropout analyses will be conducted to examine potential patterns of non-completion (Gupta, 2011). The study includes individuals with coexisting psychiatric disorders, which increases relevance to real-world clinical populations but may introduce greater heterogeneity in treatment response (Klein et al., 2013). Also several exclusion criteria, such as recent use of non-pharmacological treatments, unstable medication regimens, or co-morbid sleep disorders, were necessary to minimize confounding and improve internal validity in evaluating treatment-specific effects. However, these restrictions may reduce the external validity of findings, as such characteristics are common among individuals seeking care for insomnia. Lastly, although including participants taking stable sleep medications enhances ecological validity, variations in concurrent pharmacological treatments may influence treatment effects (Klein et al., 2013; Krämer and Köhler, 2021). This will be addressed by including sleep medication use as a covariate in ANCOVA models and through exploratory subgroup analyses.

The primary manuscript and multiple supplementary articles containing trial results have been planned for this research. These will be published in journals relevant to the specific topic. Any modifications to the current protocol will be communicated upon article publication.

## 4 Conclusion

This study will generate evidence on the efficacy of mobile dCBT-I under routine care conditions, contributing to the expanding field of digital therapeutics for sleep disorders.

If the intervention proves effective, findings from this trial may support the broader adoption of dCBT-I in clinical practice, particularly in settings with limited availability of in-person CBT—I. The study will also provide valuable data on long-term treatment effects, which may inform the development and refinement of future digital interventions.

## Abbreviations

ANCOVA: Analysis of Covariance
CBT-I: Cognitive Behavioral Therapy for Insomnia
CG: Control Group
dCBT-I: Digital Cognitive Behavioral Therapy for Insomnia
DBAS-16: Dysfunctional Beliefs and Attitudes about Sleep (16-item version)
ESS: Epworth Sleepiness Scale
GAD-7: Generalized Anxiety Disorder-7
IG: Intervention Group
ISI: Insomnia Severity Index
MMRM: Mixed-Effects Model for Repeated Measures
MFDS: Ministry of Food and Drug Safety (South Korea)
PDT: Prescription Digital Therapeutic
PHQ-9: Patient Health Questionnaire-9
RCT: Randomized Controlled Trial
SE: Sleep Efficiency
SOL: Sleep Onset Latency
SUS: System Usability Scale
TST: Total Sleep Time
WASO: Wake After Sleep Onset
WPAI-SHP: Work Productivity and Activity Impairment Questionnaire–Specific Health Problem

## CRediT authorship contribution statement

Conceptualization: DM, JK, YL. Statistical design and sample size calculation: DM, JK. Writing – original draft: DM. Writing – review and editing: DM, JK, JS, YL. Funding acquisition, project administration, and supervision: YL.

## Ethics approval and consent to participate

This study was approved by the Institutional Review Board (IRB) of each participating clinic. It will be conducted in accordance with the principles of the Declaration of Helsinki and good clinical practice. Prior to enrollment, written informed consent will be obtained from all participants. Participation in the study is voluntary, and all collected data will be handled confidentially in compliance with applicable data protection regulations.

## Trial status

Recruitment for the study is scheduled to begin in mid-February 2025, with participant enrollment taking place across six clinical sites. Data collection, including baseline, post-treatment, and follow-up assessments, is expected to be completed by March 2026.

## Funding

This study is supported by funding from the 10.13039/100008903Ministry of Health and Welfare, Republic of Korea (Grant No. RS-2023-00266002). The funders have no authority over the study design, collection, management, analysis, and interpretation of data, writing of the report, and the decision to submit the findings for publication.

## Declaration of competing interest

The authors declare the following financial interests/personal relationships which may be considered as potential competing interests: Yujin Lee reports financial support was provided by Ministry of Health and Welfare of South Korea. Daa Un Moon reports a relationship with WELT Corp. Ltd., Seoul, South Korea that includes: prior employment. Yujin Lee reports a relationship with WELT Corp. Ltd., Seoul, South Korea that includes: employment. Jeyoung Hannah Sun reports a relationship with WELT Corp. Ltd., Seoul, South Korea that includes: employment. Jeonghun Kim reports a relationship with WELT Corp. Ltd., Seoul, South Korea that includes: employment. If there are other authors, they declare that they have no known competing financial interests or personal relationships that could have appeared to influence the work reported in this paper.

## Data Availability

The datasets generated and analyzed during the study will be available from the corresponding author upon reasonable request.

## Appendix A Supplementary data

Supplementary material Image 1

## References

[bb0005] Ahn E., Baek Y., Park J.-E., Lee S., Jin H.-J. (2024). Elevated prevalence and treatment of sleep disorders from 2011 to 2020: a nationwide population-based retrospective cohort study in Korea. BMJ Open.

[bb0010] American Psychiatric Association D, Association AP (2013).

[bb0015] Andrade C. (2018). Sedative hypnotics and the risk of falls and fractures in the elderly. J. Clin. Psychiatr..

[bb0020] Backhaus J., Hohagen F., Voderholzer U., Riemann D. (2001). Long-term effectiveness of a short-term cognitive-behavioral group treatment for primary insomnia. Eur. Arch. Psychiatry Clin. Neurosci..

[bb0025] Baglioni C., Battagliese G., Feige B., Spiegelhalder K., Nissen C., Voderholzer U. (2011). Insomnia as a predictor of depression: a meta-analytic evaluation of longitudinal epidemiological studies. J. Affect. Disord..

[bb0030] Bastien C.H., Vallières A., Morin C.M. (2001). Validation of the Insomnia Severity Index as an outcome measure for insomnia research. Sleep Med..

[bb0035] Behrendt D., Ebert D.D., Spiegelhalder K., Lehr D. (2020). Efficacy of a self-help web-based recovery training in improving sleep in workers: randomized controlled trial in the general working population. J. Med. Internet Res..

[bb0040] Breslau N., Roth T., Rosenthal L., Andreski P. (1996). Sleep disturbance and psychiatric disorders: a longitudinal epidemiological study of young adults. Biol. Psychiatry.

[bb0045] Brooke J. (1996). SUS-A quick and dirty usability scale. Usab. Eval. Ind..

[bb0050] Chan A.-W., Tetzlaff J.M., Altman D.G., Laupacis A., Gøtzsche P.C., Krleža-Jerić K. (2013). SPIRIT 2013 statement: defining standard protocol items for clinical trials. Ann. Intern. Med..

[bb0055] Cho Y.W., Lee J.H., Son H.K., Lee S.H., Shin C., Johns M.W. (2011). The reliability and validity of the Korean version of the Epworth sleepiness scale. Sleep Breath..

[bb0060] Cho Y.W., Song M.L., Morin C.M. (2014). Validation of a Korean version of the insomnia severity index. J. Clin. Neurol..

[bb0065] Chung S. (2019). Korean clinical practice guideline for management of insomnia in adults. Korean Neuropsychiatr. Assoc..

[bb0070] Daley M., Morin C.M., LeBlanc M., Grégoire J.-P., Savard J. (2009). The economic burden of insomnia: direct and indirect costs for individuals with insomnia syndrome, insomnia symptoms, and good sleepers. Sleep.

[bb0075] De Crescenzo F., D’Alò G.L., Ostinelli E.G., Ciabattini M., Di Franco V., Watanabe N. (2022). Comparative effects of pharmacological interventions for the acute and long-term management of insomnia disorder in adults: a systematic review and network meta-analysis. Lancet.

[bb0080] Digital Therapeutics Alliance https://dtxalliance.org/.

[bb0085] Division M.D.R. (2023).

[bb0090] Dreyer N.A. (2018). Advancing a framework for regulatory use of real-world evidence: when real is reliable. Ther. Innov. Regul. Sci..

[bb0095] Ebert D.D., Van Daele T., Nordgreen T., Karekla M., Compare A., Zarbo C. (2018). Internet- and mobile-based psychological interventions: applications, efficacy, and potential for improving mental health. Eur. Psychol..

[bb0100] Edinger J.D., Wohlgemuth W.K., Radtke R.A., Marsh G.R., Quillian R.E. (2001). Cognitive behavioral therapy for treatment of chronic primary insomnia: a randomized controlled trial. JAMA.

[bb0105] Espie C.A., Emsley R., Kyle S.D., Gordon C., Drake C.L., Siriwardena A.N. (2019). Effect of digital cognitive behavioral therapy for insomnia on health, psychological well-being, and sleep-related quality of life: a randomized clinical trial. JAMA Psychiatr..

[bb0110] Faul F., Erdfelder E., Lang A.-G., Buchner A. (2007). G*Power 3: a flexible statistical power analysis program for the social, behavioral, and biomedical sciences. Behav. Res. Methods.

[bb0115] Fields B.G., Schutte-Rodin S., Perlis M.L., Myers M. (2013). Master’s-level practitioners as cognitive behavioral therapy for insomnia providers: an underutilized resource. J. Clin. Sleep Med..

[bb0120] Gupta S.K. (2011). Intention-to-treat concept: a review. Perspect. Clin. Res..

[bb0125] (2003). Testing the validity of the Korean SF-36 health survey. J Health Info Stat..

[bb0130] Johns M.W. (1991). A new method for measuring daytime sleepiness: the Epworth sleepiness scale. Sleep.

[bb0135] Kallestad H., Scott J., Vedaa Ø., Lydersen S., Vethe D., Morken G. (2021). Mode of delivery of Cognitive Behavioral Therapy for Insomnia: a randomized controlled non-inferiority trial of digital and face-to-face therapy. Sleep.

[bb0140] Klein J.P., Berger T., Schröder J., Späth C., Meyer B., Caspar F. (2013). The EVIDENT-trial: protocol and rationale of a multicenter randomized controlled trial testing the effectiveness of an online-based psychological intervention. BMC Psychiatr..

[bb0145] Koffel E.A., Koffel J.B., Gehrman P.R. (2015). A meta-analysis of group cognitive behavioral therapy for insomnia. Sleep Med. Rev..

[bb0150] Krämer R., Köhler S. (2021). Evaluation of the online-based self-help programme “Selfapy” in patients with unipolar depression: study protocol for a randomized, blinded parallel group dismantling study. Trials.

[bb0155] Krystal A.D., Prather A.A., Ashbrook L.H. (2019). The assessment and management of insomnia: an update. World Psychiatry.

[bb0160] Lee C., Devision D.H.D. (2020). Republic of Korea: Ministry of Food and Drug Safety.

[bb0165] Lee S.H., Shin C., Kim H., Jeon S.W., Yoon H.K., Ko Y.H. (2022). Validation of the Korean version of the Generalized Anxiety Disorder 7 self-rating Scale. Asia Pac. Psychiatry.

[bb0170] Léger D., Morin C.M., Uchiyama M., Hakimi Z., Cure S., Walsh J.K. (2012). Chronic insomnia, quality-of-life, and utility scores: comparison with good sleepers in a cross-sectional international survey. Sleep Med..

[bb0175] Lorenz N., Heim E., Roetger A., Birrer E., Maercker A. (2019). Randomized controlled trial to test the efficacy of an unguided online intervention with automated feedback for the treatment of insomnia. Behav. Cogn. Psychother..

[bb0180] Luik A.I., Kyle S.D., Espie C.A. (2017). Digital Cognitive Behavioral Therapy (dCBT) for insomnia: a state-of-the-science review. Curr. Sleep Med. Rep..

[bb0185] Malik S., Kanwar A., Sim L.A., Prokop L.J., Wang Z., Benkhadra K. (2014). The association between sleep disturbances and suicidal behaviors in patients with psychiatric diagnoses: a systematic review and meta-analysis. Syst. Rev..

[bb0190] Markwald R.R., Melanson E.L., Smith M.R., Higgins J., Perreault L., Eckel R.H. (2013). Impact of insufficient sleep on total daily energy expenditure, food intake, and weight gain. Proc. Natl. Acad. Sci..

[bb0195] Martin A., Rief W., Klaiberg A., Braehler E. (2006). Validity of the Brief Patient Health Questionnaire Mood Scale (PHQ-9) in the general population. Gen. Hosp. Psychiatry.

[bb0200] Maurer LF, Bauermann P, Karner L, Müller C, Lorenz N, Gieselmann A. Investigating the efficacy of digital cognitive behavioural therapy in comparison to a sleep-monitoring application via integrated diary and actigraphy: a randomised–controlled trial. J. Sleep Res. n/a(n/a): e14255. 10.1111/jsr.14255 PMC11744233 38895830

[bb0205] Mayer G., Jennum P., Riemann D., Dauvilliers Y. (2011). Insomnia in central neurologic diseases--occurrence and management. Sleep Med. Rev..

[bb0210] Moon D.U., Piao Z., Lee D.H., Han E. (2024). From guidelines to bedside - insomnia treatment practices in South Korea: a nationwide cohort study. Front. Psychiatr..

[bb0215] Morin C.M. (2020). Profile of Somryst prescription digital therapeutic for chronic insomnia: overview of Safety and efficacy. Expert Rev. Med. Devices.

[bb0220] Morin C.M., Vallières A., Ivers H. (2007). Dysfunctional beliefs and attitudes about sleep (DBAS): validation of a brief version (DBAS-16). Sleep.

[bb0225] Morin C.M., Bélanger L., LeBlanc M., Ivers H., Savard J., Espie C.A. (2009). The natural history of insomnia: a population-based 3-year longitudinal study. Arch. Intern. Med..

[bb0230] Morin C.M., Belleville G., Bélanger L., Ivers H. (2011). The Insomnia Severity Index: psychometric indicators to detect insomnia cases and evaluate treatment response. Sleep.

[bb0235] Neckelmann D., Mykletun A., Dahl A.A. (2007). Chronic insomnia as a risk factor for developing anxiety and depression. Sleep.

[bb0240] Ohayon M.M., Reynolds C.F. (2009). Epidemiological and clinical relevance of insomnia diagnosis algorithms according to the DSM-IV and the International Classification of Sleep Disorders (ICSD). Sleep Med..

[bb0245] Organization WH (1992).

[bb0250] Park S.-J., Choi H.-R., Choi J.-H., Kim K.-W., Hong J.-P. (2010). Reliability and validity of the Korean version of the Patient Health Questionnaire-9 (PHQ-9). Anx. Mood.

[bb0255] Qaseem A., Kansagara D., Forciea M.A., Cooke M., Denberg T.D., Physicians* CGCotACo (2016). Management of chronic insomnia disorder in adults: a clinical practice guideline from the American College of Physicians. Ann. Intern. Med..

[bb0260] Reilly M.C., Zbrozek A.S., Dukes E.M. (1993). The validity and reproducibility of a work productivity and activity impairment instrument. Pharmacoeconomics.

[bb0265] Riemann D., Perlis M.L. (2009). The treatments of chronic insomnia: a review of benzodiazepine receptor agonists and psychological and behavioral therapies. Sleep Med. Rev..

[bb0270] Riemann D., Espie C.A., Altena E., Arnardottir E.S., Baglioni C., Bassetti C.L. (2023). The European insomnia guideline: an update on the diagnosis and treatment of insomnia 2023. J. Sleep Res..

[bb0275] Riemann D., Espie C.A., Altena E., Arnardottir E.S., Baglioni C., Bassetti C.L.A. (2023). The European insomnia guideline: an update on the diagnosis and treatment of insomnia 2023. J. Sleep Res..

[bb0280] Ritterband L.M., Thorndike F.P., Gonder-Frederick L.A., Magee J.C., Bailey E.T., Saylor D.K. (2009). Efficacy of an internet-based behavioral intervention for adults with insomnia. Arch. Gen. Psychiatry.

[bb0285] Ritterband L.M., Thorndike F.P., Morin C.M., Gerwien R., Enman N.M., Xiong R. (2022). Real-world evidence from users of a behavioral digital therapeutic for chronic insomnia. Behav. Res. Ther..

[bb0290] Rossman J. (2019). Cognitive-behavioral therapy for insomnia: an effective and underutilized treatment for insomnia. Am. J. Lifestyle Med..

[bb0295] Safety MoFaD (2025).

[bb0300] Sateia M.J., Buysse D.J., Krystal A.D., Neubauer D.N., Heald J.L. (2017). Clinical practice guideline for the pharmacologic treatment of chronic insomnia in adults: an American Academy of Sleep Medicine clinical practice guideline. J. Clin. Sleep Med..

[bb0305] Schuffelen J., Maurer L.F., Lorenz N., Rötger A., Pietrowsky R., Gieselmann A. (2023). The clinical effects of digital cognitive behavioral therapy for insomnia in a heterogenous study sample: results from a randomized controlled trial. Sleep.

[bb0310] Shin H.J., Cho I.T., Choi W.S., Kim H.R., Kang M.B., Yang W.J. (2024). Digital therapeutics in Korea: current status, challenges, and future directions - a narrative review. J. Yeungnam Med. Sci..

[bb0315] Sivertsen B., Overland S., Bjorvatn B., Maeland J.G., Mykletun A. (2009). Does insomnia predict sick leave? The Hordaland Health Study. J. Psychosom. Res..

[bb0320] Sivertsen B., Øverland S., Pallesen S., Bjorvatn B., Nordhus I.H., Maeland J.G. (2009). Insomnia and long sleep duration are risk factors for later work disability. The Hordaland Health Study. J. Sleep Res..

[bb0325] Smith M.T., Perlis M.L., Park A., Smith M.S., Pennington J., Giles D.E. (2002). Comparative meta-analysis of pharmacotherapy and behavior therapy for persistent insomnia. Am. J. Psychiatry.

[bb0330] Soh H.L., Ho R.C., Ho C.S., Tam W.W. (2020). Efficacy of digital cognitive behavioural therapy for insomnia: a meta-analysis of randomised controlled trials. Sleep Med..

[bb0335] Spitzer R.L., Kroenke K., Williams J.B., Löwe B. (2006). A brief measure for assessing generalized anxiety disorder: the GAD-7. Arch. Intern. Med..

[bb0340] Thomas A., Grandner M., Nowakowski S., Nesom G., Corbitt C., Perlis M.L. (2016). Where are the behavioral sleep medicine providers and where are they needed? A geographic assessment. Behav. Sleep Med..

[bb0345] Van Straten A., van der Zweerde T., Kleiboer A., Cuijpers P., Morin C.M., Lancee J. (2018). Cognitive and behavioral therapies in the treatment of insomnia: a meta-analysis. Sleep Med. Rev..

[bb0350] Ware J.E., Sherbourne C.D. (1992). The MOS 36-item short-form health survey (SF-36). I. Conceptual framework and item selection. Med. Care.

[bb0355] World Health O (1992).

[bb0360] Wu J.Q., Appleman E.R., Salazar R.D., Ong J.C. (2015). Cognitive behavioral therapy for insomnia comorbid with psychiatric and medical conditions: a meta-analysis. JAMA Intern. Med..

[bb0365] Yaffe K., Falvey C.M., Hoang T. (2014). Connections between sleep and cognition in older adults. Lancet Neurol..

[bb0370] Yu E.-S., Ko Y.-G., Sung G.-h., Kwon J.-h. (2009). Validation of the Korean Version of Dysfunctional Beliefs and Attitudes about Sleep (K-DBAS-16). Korean J. Clin. Psychol..

[bb0375] Zachariae R., Lyby M.S., Ritterband L.M., O’Toole M.S. (2016). Efficacy of internet-delivered cognitive-behavioral therapy for insomnia - a systematic review and meta-analysis of randomized controlled trials. Sleep Med. Rev..

[bb0380] Zhang Y., Jiang X., Liu J., Lang Y., Liu Y. (2021). The association between insomnia and the risk of metabolic syndrome: a systematic review and meta-analysis. J. Clin. Neurosci..

[bb0385] van der Zweerde T., Lancee J., Ida Luik A., van Straten A. (2020). Internet-delivered cognitive behavioral therapy for insomnia: tailoring cognitive behavioral therapy for insomnia for patients with chronic insomnia. Sleep Med. Clin..

