# Supplementary material for: Applying a mobile intervention for chronic insomnia in routine care: Study protocol for a multicenter randomized controlled trial
Source: Internet Interv. 2025 Jun 20;41:100848. doi: 10.1016/j.invent.2025.100848 (PMC12240122; doi:10.1016/j.invent.2025.100848)
Supplement: Supplementary file 1 — Supplementary material [file mmc1.docx]

**Supplementary Materials for**

**Applying a mobile intervention for chronic insomnia in routine care: Study protocol for a multicenter randomized controlled trial**

**Supplementary Table 1. L**ist of Participating Clinics, Locations, and Institutional Review Board (IRB) Approvals

| **Clinic** | **Location** | **IRB No. and Institution** |
| --- | --- | --- |
| The Catholic University of Korea Seoul St. Mary’s Hospital | 222 Banpo-daero, Seocho-gu, Seoul | KC24DSDS0619  Catholic University of Korea, Seoul St. Mary’s Hospital, Institutional Review Board |
| Chungnam National University Hospital | 282 Munhwa-ro, Jung-gu Daejeon | Chungnam National University Hospital, Institutional Review Board |
| Ewha Womans University Seoul Hospital | 260 Gonghang-daero, Gangseo-gu, Seoul | 2024-09-024-004  Ewha Womans University Seoul Hospital, Institutional Review Board |
| Inje University Ilsan Paik Hospital | Juhwa-ro 170, Ilsanseo-gu, Goyang-si, Gyeonggi-do | 2024-09-004-001  Inje University Ilsan Paik Hospital, Instutional Review Board |
| Yongin Severance Hospital | 363 Dongbaekjukjeon-daero,Giheung-gu, Yongin-si, Gyeonggi-do | 9-2024-017  Yonsei University College of Medicine, Yongin Severance Hospital, Institutional Review Board |
| Yonsei Severance Hospital | 50-1 Yonsei-ro, Seodaemun-gu, Seoul | 1-2024-0052  Yonsei University Health System, Severance Hospital, Institutional Review Board |

**Supplementary Table 2.** Sample Size Calculation Using G*Power for ANCOVA


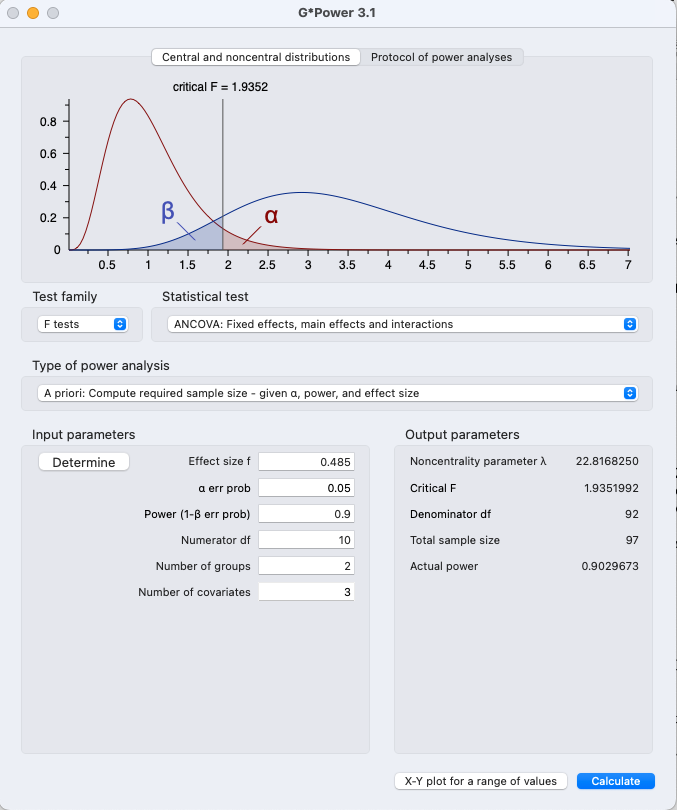


**F tests** - ANCOVA: Fixed effects, main effects and interactions

**Analysis:** A priori: Compute required sample size

**Input:** Effect size f = 0.485

α err prob = 0.05

Power (1-β err prob) = 0.9

Numerator df = 10

Number of groups = 2

Number of covariates = 3

**Output:** Noncentrality parameter λ = 22.8168250

Critical F = 1.9351992

Denominator df = 92

Total sample size = 97

Actual power = 0.9029673
